# Innovative Eco-Friendly Hydrogel Film for Berberine Delivery in Skin Applications †

**DOI:** 10.3390/molecules26164901

**Published:** 2021-08-13

**Authors:** Stefania Cometa, Maria Addolorata Bonifacio, Caterina Licini, Annalisa Bellissimo, Loris Pinto, Federico Baruzzi, Monica Mattioli-Belmonte, Elvira De Giglio

**Affiliations:** 1 Jaber Innovation s.r.l., Via Calcutta 8, 00144 Rome, Italy; stefania.cometa@jaber.it (S.C.); annalisa.bellissimo@jaber.it (A.B.); 2 Department of Chemistry, University of Bari, Via Orabona 4, 70126 Bari, Italy; maria.bonifacio@uniba.it; 3 INSTM, National Consortium of Materials Science and Technology, Via G. Giusti 9, 50121 Florence, Italy; 4 DISCLIMO, Università Politecnica delle Marche, Via Tronto 10/a, 60126 Ancona, Italy; caterina.licini@polito.it (C.L.); m.mattioli@staff.univpm.it (M.M.-B.); 5 Institute of Sciences of Food Production, National Research Council of Italy, Via G. Amendola 122/O, 70126 Bari, Italy; loris.pinto@ispa.cnr.it (L.P.); federico.baruzzi@ispa.cnr.it (F.B.)

**Keywords:** polysaccharide hydrogel, carboxymethyl cellulose, berberine, transdermal release, skin cytocompatibility, topical delivery

## Abstract

Hydrogel formulations (masks or patches, without tissue support) represent the new frontier for customizable skin beauty and health. The employment of these materials is becoming popular in wound dressing, to speed up the healing process while protecting the affected area, as well as to provide a moisturizing reservoir, control the inflammatory process and the onset of bacterial development. Most of these hydrogels are acrylic-based at present, not biodegradable and potentially toxic, due to acrylic monomers residues. In this work, we selected a new class of cellulose-derived and biodegradable hydrogel films to incorporate and convey an active compound for dermatological issues. Films were obtained from a combination of different polysaccharides and clays, and berberine hydrochloride, a polyphenolic molecule showing anti-inflammatory, immunomodulatory, antibacterial and antioxidant properties, was chosen and then embedded in the hydrogel films. These innovative hydrogel-based systems were characterized in terms of water uptake profile, in vitro cytocompatibility and skin permeation kinetics by Franz diffusion cell. Berberine permeation fitted well to Korsmeyer–Peppas kinetic model and achieved a release higher than 100 µg/cm^2^ within 24 h. The latter study, exploiting a reliable skin model membrane, together with the biological assessment, gained insights into the most promising formulation for future investigations.

## 1. Introduction

Platforms for local delivery of bioactive compounds for skin topical applications result in a significant improvement of patient compliance and adherence to therapy [1].

Nowadays, the transdermal delivery strategy is also being pursued in advanced skin care products (e.g., gels, ointments, masks), to moisturize and protect the skin while delivering bioactive compounds (i.e., antiaging, antioxidant, tissue-remodeling molecules). Indeed, Winter et al. demonstrated that keeping the wound environment moist would enhance the healing and regeneration of a lesion when compared to a dry wound environment [2]. In the case of skin injuries, proper hydration of the wound bed is compulsory to achieve complete tissue regeneration. In this context, hydrogels, with their high water content and excellent biocompatibility, are attractive tools to develop innovative skin delivery systems [3]. Within polymeric materials, eco-friendly and natural polymers represent the most promising building blocks for innovative formulations, given their biodegradable and non-toxic features [4]. Hydrogels could also be loaded with hydrophilic or hydrophobic molecules, improving their delivery through the deepest skin layers [5].

Vegetable-derived molecules, i.e., polyphenols, which are responsible of a wide range of bioactivities (antioxidant, antiaging, antimicrobial and antitumoral features), have a limited water solubility [6] and a need for suitable delivery systems to reach the target tissue. This is the case of berberine, an isoquinoline derivative exploited for its antinflammatory and antimicrobial properties: its embedding in topical products (i.e., vesicular carriers, gel delivery systems and cream formulations) has been exploited to treat several skin conditions, including wound healing [7,8]. Recently, an amino acid/berberine hydrogel and a silk-sericin derived hydrogel loaded with berberine confirmed the antimicrobial activity of berberine against Gram-positive bacteria than Gram-negative ones [9,10]. Moreover, berberine enhanced antibacterial activity of selected antibiotics against *S. aureus* strains, providing a useful strategy to overcome antibiotic-resistance issues [11].

In this work, an innovative, eco-friendly hydrogel, based on a mixture of polysaccharides and clays, has been developed to locally release berberine. Thanks to its biocompatibility, hydrophilicity, biodegradability, tissue resembling, pro-haemostatic properties and low cost [12,13,14,15], the sodium salt carboxymethyl cellulose (CMCNa) was used for the development of hydrogel films using gallium (Ga^3+^) ions as crosslinkers. This approach represents a novelty in respect to the usual-often toxic-crosslinkers, such as covalent crosslinkers (i.e., carbodiimide crosslinkers, various glycidyl ethers, etc. [16,17]) or ionic crosslinkers (aluminium salts or boric acid, borax, etc. [18,19]). Moreover, gallium was approved by the FDA to treat hypercalcemia of malignancy [20] and has recently emerged as an innovative antibacterial ion, useful in treating and preventing localized infections [21,22]. Recently, Valappil et al. proposed an ion-exchanged carboxymethyl cellulose as a source of the antibacterial ion [23].

In this work, four different formulations of a berberine-loaded hydrogel films, crosslinked with gallium ions, were synthesized and characterized. Berberine permeation through the formulations was studied by means of a Franz diffusion cell apparatus, simulating human skin in vitro permeation with the Strat-M^®^ membrane. Berberine-loaded films cytocompatibility, as well as berberine permeation, allowed selecting the system with the best potential for skin applications.

## 2. Results

### 2.1. Hydrogel Films Loaded with Berberine: Preparation and Characterization

In this study, hydrogel films were produced by a one-pot environmentally friendly process, carried out in aqueous solution and at room conditions [19]. The combination of three different polysaccharides, i.e., CMCNa, hydroxyethyl cellulose (HEC) and acetylated distarch phosphate (ADP) allowed us to obtain films, once hydrated, easily adaptable to all the parts of the body. ADP is a modified and water-soluble starch, commonly used as a food additive (E1414), used as viscosifier and texturizer in soups, sauces, gravies, bakery, and dairy products. The addition of starch or its derivatives to other CMCNa-based blends increases the biodegradation rate of CMCNa [24]. On the other hand, HEC has been considered in the polysaccharide blend here studied since it endorses intermolecular rather than intramolecular crosslinking [25]. Ga(NO_3_)_3_ was used as source of ionic crosslinking of the polysaccharide-based composite for the first time. It was hypothesized that the carboxylic acid groups present in CMCNa can be coordinated with Ga^3+^ ions, as explained by Valappil et al. [23]. Moreover, an additional step of surface crosslinking, even using Ga^3+^ ions, produced more rigid, stable, and less swellable films (HG_sx_).

As far as BERB-loaded films are concerned, two approaches to embed BERB were compared. In the first method, the molecule has been solubilized in water and then mixed in the hydrogel solution, prior to the crosslinking and drying steps (HG-BERB and HG_sx_-BERB). In the second method, the bentonite–berberine composite (BENT-BERB) was developed and then embedded into the hydrogel formulation instead of the pure berberine. The latter method could have inherent advantages for the incorporation of bioactive compounds into hydrogel matrices. Indeed, considering that berberine is a quaternary ammonium salt, an intercalation of this active compound within the bentonite layers could be allowed.

**Figure 1:**
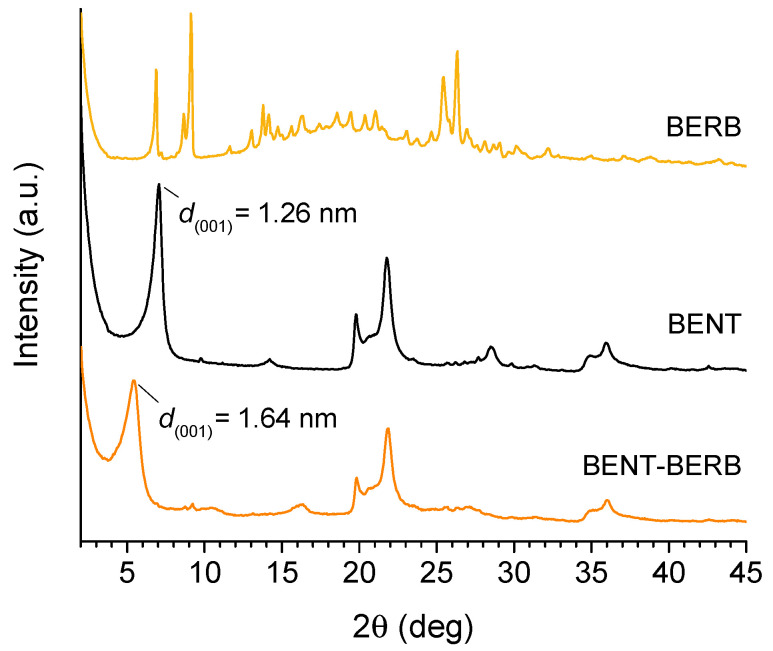
XRD analyses. X-ray diffraction patterns of berberine (BERB), bentonite (BENT) and bentonite–berberine composite (BENT-BERB).

In order to assess the intercalation of the berberine within the bentonite layers, X-ray diffraction analyses (XRD) were carried out. In Figure 1, the diffraction patterns obtained from berberine (BERB), raw bentonite (BENT) and BENT-BERB composite are reported. The profile of the BENT showed that the composition is predominantly Na-montmorillonite (MMT), characterized by an intense reflection of 001 plane at 2θ = 7.0° (d001 = 1.26 nm). The shift of the peak to lower 2θ values in BENT-BERB spectrum, corresponding to an increase of the interlayer distance from 1.26 nm (2θ = 7.0°) to 1.64 nm (2θ = 5.4°), confirmed the successful intercalation of berberine within the bentonite layers, as a result of the replacement of exchangeable cations of bentonite clays (Na+ ions) with N^+^ charged berberine. Moreover, no peaks associated to the crystalline form of berberine were observed in BERB-BENT profile. This feature indicates that the crystallization of the berberine does not occur in the experimental conditions, probably hindered by the intercalation process.

**Figure 2:**
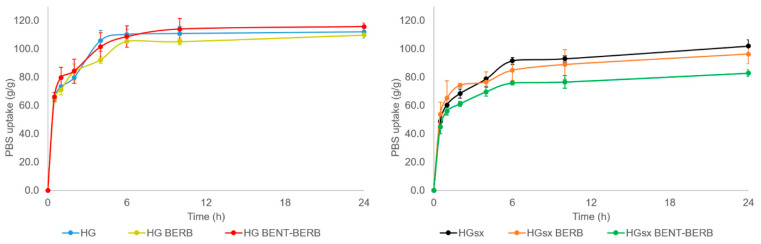
Hydrogels swelling performances. Evaluation of water uptake relevant to HG-based (left panel) and HG_sx_-based (right panel) hydrogels performed at 32 °C up to 24 h in PBS.

The films have been tested in terms of swelling performances, as shown in Figure 2. In the case of HG film, the water uptake reached a plateau after 6 h. In the case of HG_sx_ sample, the swelling was not complete after 6 h and the film continued to absorb additional liquid for up to 24 h. After 24 h, the water uptake resulted equal to 112 ± 4 and 102 ± 4 g/g for HG and HG_sx_, respectively. Therefore, the surface crosslinking resulted in a more rigid and slightly less swellable film, as expected. On the other hand, these results showed that both hydrogels fell in the range of hydrophilicity that makes them potentially suitable to promote a moist microenvironment assisting the wound healing process or required in skin care devices. The addition of BERB following the first procedure (i.e., HG-BERB and HG_sx-_BERB samples) did not significantly change the swelling performances (at 24 h, water uptake was 110 ± 1 and 96 ± 7 g/g for HG-BERB and HG_sx_-BERB, respectively). On the contrary, when the BERB-loaded films were prepared following the second procedure (i.e., HG-BENT-BERB and HG_sx-_BENT-BERB samples), a significant decrease in swelling performances relevant to HG_sx-_BENT-BERB was detected (at 24 h, water uptake was 115 ± 3 and 83 ± 2 g/g for HG-BENT-BERB and HG_sx_-BENT-BERB, respectively). This could be ascribed to the fact that, when the clay did not host the active principle, a portion of Ga^2+^ ions could preferentially interact with the anionic layers of the clay rather than crosslink the polymer network, giving rise to higher water uptake. This process could not occur when the clay hosted berberine, giving rise to lower water uptake.

Finally, the gel fraction of HG and HG_sx_ films was found to be 59.0 ± 0.5 and 65.4 ± 0.9%, clearly indicating that the surface crosslinking resulted in a more stable gel.

### 2.2. Berberine In Vitro Skin Permeation Studies

The cumulative amount of BERB, which permeated through the Strat-M^®^ synthetic membranes, was plotted against time. After 24 h, HG-BERB allowed the permeation of 137 ± 6 µg/cm^2^ of BERB, while a lower amount of the same molecule, 122 ± 8 µg/cm^2^, permeated from HG_sx_-BERB hydrogels (*p* < 0.05). The two formulations embedding BENT-BERB released a significantly lower amount of the polyphenol (91 ± 1 µg/cm^2^ for HG-BENT-BERB and 72 ± 6 µg/cm^2^ for HG_sx_-BENT-BERB, *p* < 0.001) with respect to the other films in which berberine was not included in the BENT. Furthermore, the analysis of BERB retained within the Strat-M^®^ membranes for each of the four hydrogel types was performed. The membranes exploited for HG-BERB and HG_sx_-BERB hydrogels permeation study did not retain BERB, while 3.7 ± 0.4 µg/cm^2^ and 8.3 ± 0.6 µg/cm^2^ of BERB were retained within the membranes used to test HG-BENT-BERB and HG_sx_-BENT-BERB samples, respectively. Therefore, since the Strat-M^®^ membrane ability to hinder BERB permeation was a common experimental parameter for all the hydrogels tested, their differential BERB release kinetics might explain the observed data.

**Figure 3:**
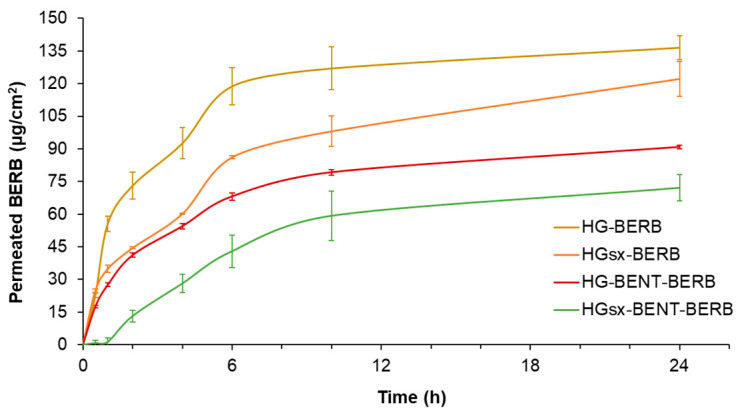
Skin permeation of BERB from the hydrogel formulations. BERB amount permeated through Strat-M^®^ membranes within 24 h. Experiments were performed in triplicate.

As shown in Figure 3, BERB permeation kinetic was dramatically delayed by the presence of the surface crosslinker in both the systems. Indeed, after 1 h, 56 ± 3 µg/cm^2^ of BERB permeated from HG-BERB hydrogel, while the surface crosslinked formulation released 35 ± 2 µg/cm^2^ of BERB, corresponding to the 41 and 30% of the cumulative BERB permeated in 24 h, respectively. Similarly, HG_sx_-BENT-BERB more slowly released the polyphenol in comparison with the HG-BENT-BERB system, which released 28 ± 1 µg/cm^2^ of BERB. Indeed, BERB release of HG_sx_-BENT-BERB after one hour of incubation was negligible.

These results encourage the use of the hydrogels here developed for those applications in which a controlled-release profile is required. In this respect, Vanti et al. developed a persistent delivery system based on escinosomes loaded with BERB. Their formulation released 40% of BERB within the first 30 min through a synthetic membrane simulating the skin’s stratum corneum. Since Vanti et al. proposed their formulation as a persistent delivery system [7], also the films herein developed could be exploited similarly. In particular, HG_sx_-BERB film showed a controlled BERB release, while achieving a noteworthy BERB permeation within 24 h. Indeed, the same BERB amount is considered effective to elicit topical beneficial effects [26]. Moreover, in comparison with the previously described system [7], BERB permeation was herein tested through Strat-M^®^ membranes, considered a reliable model of human skin double layer, useful to consistently predict the in vivo permeation behavior of active pharmaceutical ingredients [27].

Furthermore, BERB permeation from the four hydrogels through the Strat-M^®^ membrane was non-linear. Therefore, to assess the potential mechanisms of BERB release occurring from the hydrogels, three kinetic models (i.e., zero order, Higuchi and Korsmeyer–Peppas models) were used to fit the experimental data, comparing their correlation coefficients. As reported in Table 1, the highest R^2^ values were obtained from the Korsmeyer–Peppas model. The latter was specifically built to focus on drug release from polymeric systems, providing insights into diffusion-based and polymeric relaxation mechanisms [28].

The data in Table 1 suggest that BERB permeation from HG_sx_-BERB hydrogel was mainly driven by Fickian diffusion. Indeed, applying the Korsmeyer-Peppas model to fit the data, an *n* value of 0.37 was obtained, whereas it reached 0.68 for HG-BERB. Thus, the latter hydrogel allowed a BERB release, which might involve further mechanisms of BERB permeation, beyond diffusion (i.e., polymeric chains relaxation). As far as the systems embedding BERB into the bentonite clay, the results reported in Table 1 indicate that HG-BENT-BERB triggered a diffusion-driven permeation kinetic, whereas HG_sx_-BENT-BERB hydrogel led to a BERB release combining diffusive processes with polymer relaxation and erosion. Under the experimental conditions set up, after 24 h BERB permeation achieved was around 50 µM (17 μg/mL). Literature on BERB inhibitory activity against skin related microorganisms is controversial, but BERB-loaded hydrogels could exert some antimicrobial effect. Indeed, 16 μg/mL of BERB inhibited the growth of coagulase-negative *S. capitis* ATCC 35661, while 32 μg/mL were needed against *S. epidermidis* ATCC 12228 [11]. Conversely, other microorganisms were less susceptible to BERB, i.e., *S. aureus*, *B. subtilis*, *E. coli*, *M. smegmatis* and *C. albicans*, according to the National Committee of Clinical Laboratory Standards [29]. Moreover, BERB derivatives were also synthetized to exert higher MexXY inhibitory activities, lowering the MICs for two *P. aeruginosa* strains from 512 µM up to 64 µM [30]. Therefore, combining the obtained data with literature findings on berberine skin benefits, HG_sx_ films were selected for in vitro biological assessment.

### 2.3. Cytotoxicity Assessment and Stress Fiber Formation

**Figure 4:**
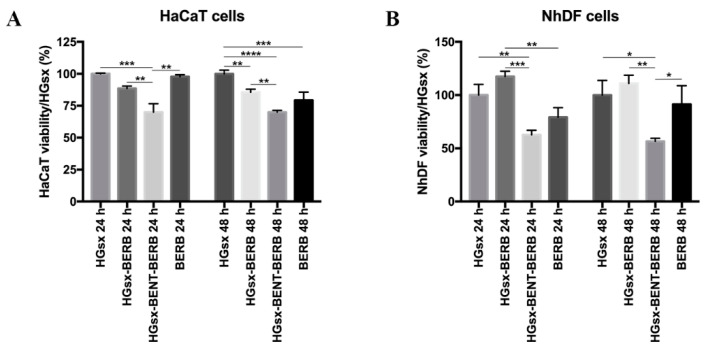
XTT assay. HaCaT (**A**) and NhDF (**B**) cells were treated with HG_sx_, HG_sx_-BERB, HG_sx_-BENT-BERB conditioned media and medium with 50 µM Berberine. (* *p* ≤ 0.05; ** *p* ≤ 0.01; *** *p* ≤ 0.001; **** *p* ≤ 0.0001).

Cells were treated up to 48 h with the media conditioned by HGsx and HGsx-BERB, HGsx-BENT-BERB and medium containing 50 µM BERB (corresponding to 17 µg/mL, the maximum amount of polyphenol released) to assess cell viability. At 24 h, XTT assay showed a good viability in human epidermal keratinocytes (HaCaT) cells treated with HGsx, HGsx-BERB and BERB conditioned media, whilst a significant reduction was observed in HGsx-BENT-BERB group. At 48 h, the trend was confirmed for HGsx, HGsx-BERB and HGsx-BENT-BERB, whereas cells treated with BERB exhibited a slight reduction in viability (Figure 4A). At 24 h, Normal Human Dermal Fibroblasts (NhDF) cells displayed low viability with HGsx-BENT-BERB and 50 µM BERB treatments, compared to cells treated with HGsx and HGsx-BERB conditioned media that exerted a good effect on cells. At 48 h from treatment, similar data were observed, although a slight increase in fibroblasts treated with BERB medium was found (Figure 4B).

**Figure 5:**
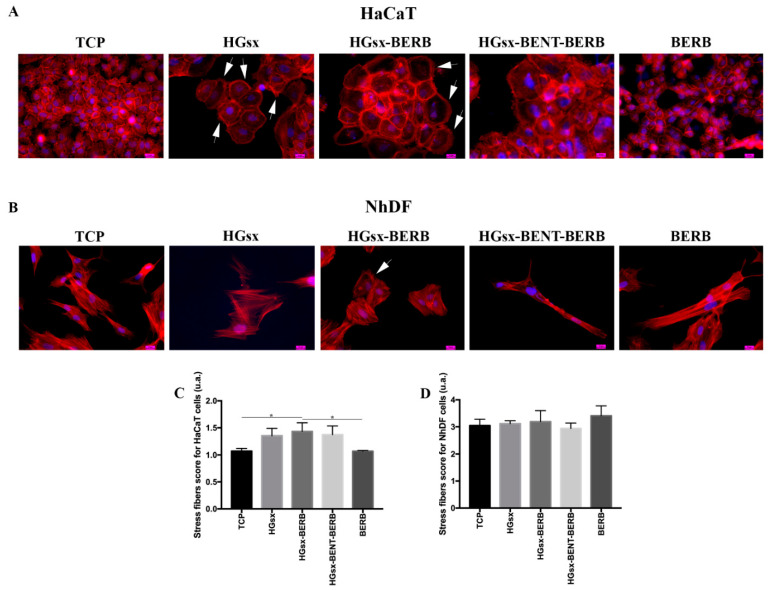
Morphological and stress fibers evaluations. Representative images of actin cytoskeleton immunofluorescence detection in HaCaT (**A**) and NhDF (**B**) cultured in the different conditions. 40× magnification. Scale bar 10 µm. White arrows indicate migration features in cells: lamellipodia and filopodia in HaCaT cells (**A**) and actin polarization at the cellular front in NhDF cells (**B**). (**C**) Stress fibers score for HaCaT cells: significant differences between TCP and HGsx-BERB, and HGsx-BERB and BERB were found. (* *p* ≤ 0.05) (**D**) Stress fibers score for NhDF cells.

F-actin staining highlights cell morphology, providing further information on cell status. After 48 h, HaCaT cells cultured with or without 50 µM BERB showed similar morphology, with cortical actin disposition emphasizing the cell–cell connection typical of keratinocytes. Differently, in HaCaT cells treated with HGsx, actin stress fibers, lamellipodia and filopodia occurred at the expense of cortical actin filaments, showing cell–cell separation and total or partial loss of intercellular junctions. These features are suggestive of a migratory condition [31]; an increase in cell dimension was also detected. Similar features were detected in HaCaT treated with HGsx-BERB conditioned media, even though at a lower extent (Figure 5A,C). HaCaT cultured in HGsx-BENT-BERB conditioned medium exhibited a slight cortical staining and a morphology superimposable to CM control, although with some cells increased in dimension. NhDF cultured with or without 50 µM BERB and HGsx-BENT-BERB showed a spindle-shaped morphology at 48 h, whereas cells cultured in HGsx-conditioned medium displayed a more flattened cuboidal shape. NhDF cells treated with HGsx-BERB-conditioned medium presented an elongated morphology similar to CM control, even if they were slightly wider in shape. Few cells showed assembled actin at the cell front, forming a polarized border with lamellipodia and filopodia typical of migratory fibroblasts [32]. Cells of all the groups exhibited well-defined actin filaments along the entire cell length with no significant differences in stress fibers score. (Figure 5B,D) These findings suggested that BERB did not affect fibroblast morphology, although it stimulated migratory aspects in certain NhDF cells.

Wound healing is a complex process leading to the repair of an injury that occurred in the epithelium or the deepest subcutaneous tissues and involves both keratinocytes and fibroblasts. In this respect, our results indicated a positive effect of HGsx and HGsx-BERB on HaCaT and NhDF.

In wound repair, the epithelialization stage starts a few hours after the damage and the epidermis begins thickening within 24 h of damage. Before leaving the wound edge, keratinocytes disassemble cell–cell and cell–extracellular matrix contacts to reach the uncovered area and close the gap, whilst keratinocytes behind the migrating tongue start to proliferate [33,34]. Our results showed that at 48 h, HaCaT cells treated with HGsx- and HGsx-BERB-conditioned media are capable of assuming a migratory feature, as evidenced by the presence of actin stress fibers, lamellipodia and filopodia as well as cell–cell separation and total or partial loss of intercellular junctions. We also observed an optimal viability in keratinocytes with these treatments, suggesting that HGsx films, with and without BERB, could stimulate keratinocyte activation, thus improving the re-epithelialization during the wound healing process. At the wound site, an increase of fibroblast number usually occurs by both migration and proliferation 48 h later of the injury and achieves peak number on the seventh day [34]. Here, we observed a positive viability in NhDF cells at 48 h-treatment with HGsx- and HGsx-BERB-conditioned medium. This suggests the cytocompatibility of the two systems and that the CMCNa-based material is capable of hampering a possible detrimental effect of BERB observed in NhDF cells. Furthermore, some fibroblasts treated with HGsx-BERB-conditioned medium reorganized F-actin fibers to facilitate cell mobility, suggesting a possible stimulation of fibroblasts migration toward the wound site during the repair process. Overall, we can speculate that the HGsx-BERB system could exert a beneficial effect on wound repair favoring re-epithelialization and triggering successive fibroblast migration as in the physiological process [34].

## 3. Materials and Methods

### 3.1. Materials

Carboxymethyl cellulose sodium salt, CMCNa (MW 700 kDa, DS 0.7, pharmaceutical grade) and hydroxyethyl cellulose, HEC (MW 250 kDa MS 2, viscosity 80–125 cm/s, food grade) were purchased from Eigenmann e Veronelli S.p.A. (Milan, Italy). Bentonite (BENT) was supplied by Dal Cin S.p.A. Sesto San Giovanni (Milan, Italy) and Laponite^®^ RD was supplied by Byk (Altana Group). Acetylated distarch phosphate (ADP) was supplied by Romana Chimici S.p.A. (Palo del Colle, Bari, Italy). Berberine hydrochloride from *Berberis aristata* dry extract at 98% (BERB) was purchased from Farmalabor s.r.l. (Canosa di Puglia, Apulia, Italy). Gallium nitrate (GaNO_3_)_3_ and ethanol, as well as ultrapure water and methanol (all HPLC grade), were purchased from Sigma Aldrich (Milan, Italy).

### 3.2. Hydrogel Films Preparation

Hydrogel films were prepared exploiting the method described in the patent of Cometa et al. [19], opportunely modified. Briefly, CMCNa, HEC and ADP polymers, in the ratio 3:1:0.12 *w*/*w*, were mixed to bentonite and Laponite^®^ powders (in the ratio 10:1, *w*/*w*). In this mixture, the clays were 10% *w*/*w* of the content of the polymers. The mixture was dispersed in distilled water (in the range 2% *w*/*v*) containing Ga(NO_3_)_3_ at 2% *w*/*w* respect to the content of the polymers, until the complete homogenization and hydration of polymers and clays mixture. The smooth and homogeneous film-forming solution was transferred in an ultra-sonication bath to remove air bubbles and then casted on Petri dishes (diameter 17 cm) and dried at 80 °C for 3 h. The obtained film was herein indicated as HG. To obtain more stable material, the films underwent a second crosslinking procedure, employing a surface crosslinking solution containing Ga(NO_3_)_3_ at 10% *w*/*w* respect to the film weight. The solution was sprayed over both the surfaces of the film, and the latter was successively dried at 80 °C for 1 h. This surface cross-linked film was indicated as HG_sx_.

For BERB-loaded hydrogel films, two different procedures were tested. In the first one, the polyphenolic compound was added to the film formulation, solubilizing BERB in a part of the distilled water employed in the hydrogel preparation (HG-BERB). As previously reported, a film with an additional surface crosslinking (HGsx-BERB) was produced.

In the second procedure, the BENT-BERB composite was prepared. Briefly, berberine was solubilized in hot water (80 °C), followed by addition of the clay in a ratio 1.1:1 *w*/*w* BERB/BENT. Then, the suspension was sonicated for 10 min and dried at 60 °C until constant weight. The obtained BENT-BERB composite was used in the hydrogel film preparation (HG-BENT-BERB). Additionally, in this procedure, a sample with additional surface-crosslinking was prepared (HGsx-BENT-BERB). Details relevant to the six films prepared, were reported in Table 2.

### 3.3. XRD Analysis of the BENT-BERB Composite

The intercalation of the berberine into the bentonite clay was evaluated by means of X-ray diffraction (XRD) analysis. The patterns were collected by a PANanalytical X’Pert powder diffractometer operating with a Bragg–Bentano θ/θ configuration reflection mode. The instrument, equipped by a Ni-filter Cu-Kα source (λ = 1.518 Å), was run at 40 kV and 40 mA and all the measurement were recorded in the 2θ range of 2–45° at room temperature.

### 3.4. Hydrogel Film Swelling and Gel Fraction

Hydrogel film samples were cut into square pieces (1 cm × 1 cm), accurately weighed (m_i_^d^) and successively placed in tea bags. The tea bags containing samples were sealed and immersed in phosphate buffer solution (PBS, pH 7.4) at 32 °C to determine the swelling kinetics up to 24 h. All the specimens were weighted prior (m_i_^0^) and after each time point (m_i_^t^), subtracting the weight of empty wet tea bag. The water uptake (expressed as grams of PBS per gram of dry film) was calculated by the following formula:

Water uptake = [((m_i_^t^ − m_b_^t^) − m_i_^0^)/m_i_^d^] (1)

To determine the gel fraction, dry HG and HGsx film samples (1 cm × 1 cm) were weighted accurately and then placed in distilled water and incubated at 50 °C for 24 h for removal of soluble (i.e., not crosslinked) parts. The insoluble gel obtained was dried in the oven at 70 °C until constant weight. The gel fraction was determined from the following formula:

Gel fraction (%) = W_f_/W_i_ × 100 (2)

where W_f_ and W_i_ are the weight of insoluble dry gel and the initial weight of the dry film.

### 3.5. Berberine In Vitro Skin Permeation Studies

A jacketed Franz diffusion cell (PermeGear Inc., SES GmbH, Bechenheim, Germany) was exploited to assess in vitro skin permeation of BERB eluting from hydrogel films. BERB-loaded hydrogel films were placed in the cell donor compartment and hydrated with 1 mL of PBS (pH 7.4). An O-ring joint kept the film (0.6 cm^2^) on the synthetic Strat-M^®^ membrane (Merck KGaA, Darmstadt, Germany), characterized by skin-like porosity, diffusivity and composition. The whole assembly was fixed with a stainless-steel clamp to maintain the tight connection between the donor and receptor compartments. The Franz cell receptor chamber was filled with 5 mL of PBS (pH 7.4) and continuously stirred on an ATE magnetic stirrer (VELP Scientifica Srl, Usmate, Italy). The temperature was kept constant at 32.00 ± 0.03 °C with a CD-B5 heating circulator bath (Julabo GmbH, Seelbach, Germany). At predetermined time points (30 min, 1, 2, 4, 6, 10, and 24 h) PBS aliquots of 200 µL were withdrawn, replaced with fresh PBS and analyzed by HPLC (Prominence Series 20 with SPD-M20A PDA detector, Shimadzu, Milan, Italy) for BERB content, adapting the method previously described by Shigwan et al. [35]. Each sample was tested three times and data reported as mean ± standard deviation. A Shim-Pack GIST C18-AQ column (150 mm × 4.6 mm, 5 μm Shimadzu) was eluted in isocratic mode at 30 °C, 40% acetonitrile, 60% water with 1% trifluoroacetic acid. The effluent was monitored at 344 nm. The mobile phase flow rate was kept at 1 mL/min, and samples were injected through a 20 µL injection loop. LabSolutions software was exploited to build a calibration curve (r^2^ 0.999) with the standard compound dissolved in mobile phase at four concentrations (1, 5, 25, 100 µg/mL).

Additionally, at the end of the experiments, the Strat-M^®^ membrane was placed in the HPLC mobile phase overnight at 25 °C to extract and quantify the retained BERB.

Different kinetic models were studied to describe the experimental release kinetics relevant to the four hydrogels prepared. Firstly, a zero-order kinetic model was considered since it is useful to describe slowly releasing systems [36]. This model can be summarized by the following equation:

Q_t_ = Q_0_ + k_0_ t (3)

in which Q_t_ represents the berberine content released at time t, while Q_0_ corresponds to the starting berberine concentration in solution (Q_0_ = 0) and k_0_ is the zero-order release constant.

Secondly, the Higuchi model was studied to predict the release of berberine, a quite-hydrophilic molecule, as per the Equation (4):

Q_t_ = k_H_ √t (4)

in which Q_t_ represents the berberine content at time t, while k_H_ is the Higuchi dissolution constant [37].

Thirdly, the Korsmeyer–Peppas model was investigated, to describe berberine release from a polymeric system able to swell and degrade in aqueous media [28]. The exploited equation was the following:

M_t_/M_∞_ = k_r_ t^n^ (5)

in which M_t_/M_∞_ indicates the ratio between the berberine content at time t and the total berberine released, k_r_ represents the release constant and n is the diffusion descriptor.

### 3.6. Cell Culture and Cytocompatibility Assessment

#### 3.6.1. Cell Culture

Human immortalized keratinocytes (HaCaT) and normal human dermal fibroblasts (NhDF) were cultured in High Glucose Dulbecco’s Modified Eagle Medium (HG-DMEM; Corning Inc., Corning, NY, USA), supplemented with 10% fetal bovine serum (Corning Inc.), 1% L-glutamine (Thermo Fisher Scientific, Waltham, MA, USA) and 1% penicillin/streptomycin (Thermo Fisher Scientific) (henceforward referred as CM), and maintained in a humidified incubator at 37 °C with 5% CO_2_. All the experiments were conducted in triplicate.

#### 3.6.2. BERB and Hydrogels Effect on Cell Viability

To assess the effect of the developed material on cell viability, HGsx, HG_sx_-BERB and HG_sx_-BENT-BERB were incubated at 37 °C for 24 h in CM, to obtain a conditioned medium. HaCaT and NhDF cells were seeded into 96 well plates at cell density of 5 × 10^4^ and 8 × 10^3^ cells/cm^2^, respectively, and cultured up to 48 h in the three conditioned media, in a medium containing dissolved BERB at the maximum amount (50 ∝M) released by the hydrogel, and in CM. Increasing concentrations of Berberine (from 12.5 µM to 100 µM) were also tested (data not shown). Cell viability was then examined by sodium 3′-[1-(phenylaminocarbonyl)-3,4-tetrazolium]-bis(4-methoxy6-nitro)benzene sulfonic acid hydrate (XTT) colorimetric assay (Sigma-Aldrich, Milan, Italy) at 24 and 48 h, according to the manufacturer’s instruction. Absorbance at 555 nm with 655 nm as reference wavelength was read using MultiskanGO plate reader (Thermo Fisher Scientific).

XTT data are expressed as percentage with respect to HGsx.

#### 3.6.3. Cell Morphology and Stress Fiber Formation

HaCaT and NhDF cells were seeded on 4-well chamber slides at a cell density of 5 × 10^4^/cm^2^ and 8 × 10^3^/cm^2^, respectively, and treated as stated above.

Cells were fixed with 4% paraformaldehyde in PBS pH 7.4 (Santa Cruz Biotechnology, Dallas, TX, USA) at 4 °C for 30 min, washed three times in PBS and permeabilized with 0.1% Triton X-100 in PBS at RT for 30 min. Cells were then incubated with TRITC-labelled phalloidin (Thermo Fisher Scientific, dilution 1:100) for 45 min at RT to highlight F-actin fibers, and with Hoechst 33342 (Thermo Fisher Scientific, dilution 1:10,000) to stain cell nuclei. Slides were mounted with Vectashield mounting medium and analyzed under fluorescent microscope Eclipse 600 (Nikon, Milan, Italy). NIS-Elements microscope imaging software (Nikon) was used to capture images.

To measure stress fibers degree, four images at 40× magnification for HaCaT and 20× magnification for NhDF were taken and at least 60 cells were examined, and a five-point scoring system was used, according to the following criteria: (1) little or no determined F-actin stress fiber formation and mainly cortical actin; (2) thin, short F-actin filaments interesting at least 25% of the cell; (3) moderate F-actin stress fiber with thicker stress fibers occupying at least 50% of the cell; (4) thick and well-defined stress fibers with extensive stress fiber traversing the full width of the cell; (5) the entire cell is heavily packed with thick stress fibers, most crossing the width of the cell [38].

### 3.7. Statistical Analyses

The statistical analysis was performed using GraphPad Prism 7 (GraphPad Software, San Diego, CA, USA). In vitro permeation results, as well as viability and morphological data, were analyzed by Mann–Whitney and ANOVA tests. After ANOVA, multiple comparisons among the groups were analyzed by Tukey’s test. Statistical significance was considered at *p* < 0.05.

## 4. Conclusions

In this study, a class of novel, ecofriendly and cytocompatible hydrogel films, loaded with berberine, were developed. Gallium was chosen in order to avoid the traditional and often toxic crosslinkers employed for CMC-based hydrogels. The optimization of the hydrogel composition and crosslinking procedure allowed influencing the gel stiffness, as well as its water uptake. The prepared films resulted as nontoxic for both fibroblast and keratinocytes, inducing migratory features in HaCaT cells, advantageous to support the wound healing process in vivo. The in vitro berberine permeation kinetics through an artificial skin membrane, together with the cytotoxicity studies, allowed pointing out the most suitable system for interesting investigations for skin treatments.

## Figures and Tables

**Table 1:** Kinetic modelling of BERB release. Application of three different models to fit in vitro permeation data.

Formulation	Zero Order		Higuchi		Korsmeyer-Peppas		
	K_0_	R^2^	K_H_	R^2^	K_r_	N	R^2^
HG-BERB	5.84	0.759	26.5	0.917	34.6	0.68	0.981
HG_sx_-BERB	5.37	0.876	23.4	0.978	27.7	0.37	0.999
HG-BENT-BERB	5.58	0.829	24.8	0.959	30.1	0.51	0.997
HG_sx_-BENT-BERB	5.15	0.899	21.1	0.965	6.75	1.23	0.994

**Table 2:** Hydrogel films composition.

Film	Percentage of Each Component to the Total Mass of Dried Film (%)								
	CMCNa	HEC	ADP	BENT	LAP	BERB	BENT-BERB	Bulk Crosslinker	Surface Crosslinker
HG	64.6	21.5	2.6	8.6	0.9	--	--	1.8	--
HG_sx_	58.7	19.6	2.3	7.8	0.8	--	--	1.6	9.1
HG-BERB	58.8	19.6	2.4	7.8	0.8	8.9	--	1.6	--
HGsx-BERB	53.5	17.8	2.1	7.1	0.7	8.1	--	1.5	9.1
HG-BENT-BERB	58.8	19.6	2.4	--	0.8	--	16.9	1.6	--
HGsx-BENT-BERB	53.5	17.8	2.1	--	0.7	--	15.3	1.5	9.1

## Data Availability

The data presented in this study are available herein.

## Sample Availability

Samples of the developed hydrogels are available from the authors.
